# Immunomodulatory Mechanisms of Tea Leaf Polysaccharide in Mice with Cyclophosphamide-Induced Immunosuppression Based on Gut Flora and Metabolomics

**DOI:** 10.3390/foods13182994

**Published:** 2024-09-21

**Authors:** Qiaoyi Zhou, Jinjing Gao, Xueyan Sun, Junyuan Du, Zhiyi Wu, Dongxia Liang, Caijin Ling, Binghu Fang

**Affiliations:** 1 Guangdong Provincial Key Laboratory of Tea Plant Resources Innovation and Utilization, Tea Research Institute, Guangdong Academy of Agricultural Sciences, Guangzhou 510640, China; zhouqyi@foxmail.com (Q.Z.); liangdx3407@163.com (D.L.); 2 National Reference Laboratory of Veterinary Drug Residues, College of Veterinary Medicine, South China Agricultural University, Guangzhou 510640, Chinawuzhiyi202302@163.com (Z.W.)

**Keywords:** tea polysaccharide, immune regulation, gut microbiota flora, metabolomic

## Abstract

Tea polysaccharides (TPSs) are receiving increasing attention because of their diverse pharmacological and biological activities. Here, we explored the immunoregulatory mechanisms of TPSs from fresh tea leaves in a mouse model of cyclophosphamide (CTX)-induced immunosuppression in terms of gut microbiota and metabolites. We observed that TPSs significantly increased the body weight and alleviated CTX-induced thymus atrophy in the immunosuppressed mice; they also increased the plasma levels of immunoglobulins A and M, interleukin (IL) 1β, IL-6, inducible nitric oxide synthase, and tumor necrosis factor α. Furthermore, we conducted 16S rDNA sequencing of cecal contents, resulting in the acquisition of 5008 high-quality bacterial 16S rDNA gene reads from the sequencing of mouse fecal samples. By analyzing the data, we found that TPSs regulated the gut microbiota structure and diversity and alleviated the CTX-induced dysregulation of gut microbiota. The colonic contents of mice were subjected to analysis using the UPLC-Q-TOF/MS/MS technique for the purpose of untargeted metabolomics. In the course of our metabolite identification analysis, we identified a total of 2685 metabolites in positive ion mode and 1655 metabolites in negative ion mode. The analysis of these metabolites indicated that TPSs improved CTX-induced metabolic disorders by regulating the levels of metabolites related to tryptophan, arginine, and proline metabolism. In conclusion, TPSs can alleviate CTX-induced immunosuppression by regulating the structural composition of gut microbiota, indicating the applicability of TPSs as novel innate immune modulators in health foods or medicines.

## 1. Introduction

Plant polysaccharides (PPSs) are crucial, naturally occurring active ingredients which can be obtained from a wide range of plant species; they are safe and have low toxicity levels [1]. Polysaccharides are a class of macromolecular compounds which are composed of more than 10 monosaccharides linked by glycosidic bonds. Numerous studies have demonstrated that PPSs have a variety of biological activities, including antioxidant [2], immune regulation [3], hypoglycemic [4], and anti-tumor [5].

In recent years, there has been a growing scholarly interest in the immunomodulatory properties of PPSs. Studies have indicated that PPSs can regulate the growth and development of immune organs and enhance the body’s resistance to bacteria and viruses [6]. Polysaccharides are key factors in cell surface signal recognition, intercellular signaling, and immune-related reactions. They can initiate immune responses by interacting with receptors such as dectin-1, toll-like receptor (TLR), or mannose receptor (MR) on mononuclear phagocytes and antigen-presenting cells. In addition, polysaccharides also activate immune responses through complement receptor type 3 (CR3) on granulocytes, neutrophils, and natural killer cells [3]. Furthermore, the elucidation of the connections between gut microbiota and host immunity has led to an increased focus on the immunoregulatory mechanisms of PPSs that target gut microbiota. Research indicates that the upper gastrointestinal tract may find PPSs are difficult to digest; however, they can reach the large intestine, where they interact with intestinal microbes to enhance overall health by modulating intestinal flora, metabolites, and the morphology of intestinal tissues. Specifically, polysaccharides can enhance the diversity of intestinal flora, selectively promote the proliferation of beneficial bacteria, and function as prebiotics [7]. Additionally, these polysaccharides can modulate the immune response by altering the composition and abundance of intestinal flora, strengthening intestinal barrier function, and facilitating the production of short-chain fatty acids (SCFAs) [8]. Numerous studies have indicated that the bioactivities of polysaccharides are dependent on molecular weight (MW), monosaccharide composition, glycosidic bond configurations, and the presence of polysaccharide conjugates [9,10]. For instance, research has demonstrated that the arabinogalactan backbone is prevalent in many plant polysaccharides that possess immunomodulatory properties, with those containing higher concentrations of arabinose and galactose potentially exhibiting enhanced immunomodulatory effects. Consequently, the biological activity of polysaccharides is primarily influenced by their chemical composition and structural characteristics [11]. However, the structure–activity relationship of polysaccharides remains inadequately explored, indicating a need for further investigation in this area.

Tea, an agricultural commodity derived from the fresh leaves and buds of the Camellia sinensis plant, has a rich historical background in both dietary and medicinal applications. As reported by the International Tea Commission, global tea consumption surpassed 5.8 million metric tons in 2019 [12]. The rising demand for tea and its derivative products has led to a rapid increase in the generation of tea waste—primarily consisting of unharvested and pruned fresh tea leaves—during the tea production process. This has resulted in large biomass losses and increased environmental pressure. Tea contains various functional components, mainly including polyphenols, polysaccharides, alkaloids, and amino acids. As such, it can be used as a source of substances with high efficiency, easy availability, and natural functional action components [13,14]. Tea polysaccharides (TPSs) are a type of main active component in tea that have several biological functions, including antioxidative [2], immune-enhancing [15], hypoglycemic [4], and anticancer activities [16]. Considerable TPS research has been conducted not only in China but also internationally, but the material basis and mechanism of action of TPSs remain unknown; this has remained the main reason behind the limited development of TPS-based interventions.

The immune system is essential for the protection of the host against pathogens and exogenous substances [17,18]. Interactions between the innate and adaptive immune systems are essential for the preservation of host health; therefore, dysfunction in or compromise of the immune system allows for the development of various infections and diseases [19,20]. Cyclophosphamide (CTX) is an effective immunosuppressive agent that is widely used for treating various immune disorders and malignancies. CTX inhibits the proliferation of immune cells, such as lymphocytes and macrophages, or the normal development of immune organs, thereby reducing cellular and humoral immune responses [21]. When used over the long term or at high doses, CTX can engender damage to the immune system and mucosal barrier of the intestines, disrupting the gut microbiota’s structural composition [22]. Therefore, CTX is commonly used to establish immunosuppressed murine models in studies investigating the immunoregulatory activities of drugs.

To further explore mechanisms underlying the immunoregulatory activities of TPSs, we extracted TPSs from fresh tea leaves and established a mouse model of CTX-induced immunosuppression. Next, we assessed the effects of TPSs on immune organ and blood indexes, plasma immunoglobulin (Ig) levels, and plasma and spleen cytokine levels in the immunosuppressed mice. We also used 16S rDNA high-throughput technology, ultraperformance liquid chromatography coupled with a hybrid quadrupole orthogonal time-of-flight tandem mass spectrometry (UPLC-Q-TOF/MS/MS), and other related molecular biological methods for assessing the effects of TPSs on gut microbiota and metabolites.

## 2. Materials and Methods

### 2.1. Materials and Animals

We procured fresh Taicha No. 12 tea leaves from Zijin County Zilong Agricultural Development Co., Ltd. (Zijin County, Heyuan City, China), CTX and levamisole hydrochloride (LH) from Sigma Chemical Co. (St. Louis, MI, USA), a hematoxylin and eosin (H&E) staining solution kit from Pinofei Biotech (Wuhan City, China), commercial enzyme-linked immunosorbent assay (ELISA) kits for various mouse proteins [namely IgA, IgM, inducible nitric oxide (NO) synthase (iNOS), tumor necrosis factor (TNF) α, interleukin (IL) 6, and IL-1β] from Jiangsu Enzyme Immunoassay Industry (Yancheng, China). All other chemicals were of analytical grade quality.

Male BALB/c mice (age: six weeks; weight: 20 ± 2 g) were purchased from Liaoning Changsheng Biotechnology [license number: SCXK (LIAO) 2020-0001]. The protocols for all animal experiments were approved by the Experimental Animal Ethics Committee of South China Agricultural University (approval number: 2023B160).

### 2.2. TPS Extraction and Preparation

Polysaccharides are categorized as polar macromolecular compounds that demonstrate solubility in hot water while remaining insoluble in organic solvents, such as ethanol. This property facilitates their extraction through water extraction followed by alcohol precipitation, a well-established technique that has been extensively utilized in the relevant field [23]. Consequently, we implemented this method for the initial extraction of crude polysaccharides. This was subsequently followed by deproteinization using the Sevag method, petroleum ether fat removal, and the elimination of low-molecular-weight substances via the macroporous adsorption resin method, ultimately resulting in the isolation of TPSs, which was in accordance with our previous studies [24]. The total carbohydrate content, protein content, and MW of TPSs were 68.89%, 25.67%, and 478.75 kDa, respectively. The TPSs were composed of Fuc, Rha, Ara, Gal, Glc, Xyl, Man, Gal-UA, and Glc-UA at a molar ratio of 4.41:3.39:1.59:1.00:0.76:0.63:0.29:0.18:0.13 [24].

### 2.3. Animal Grouping Based on TPS Administration and Mouse Model Establishment

**Figure 1:**
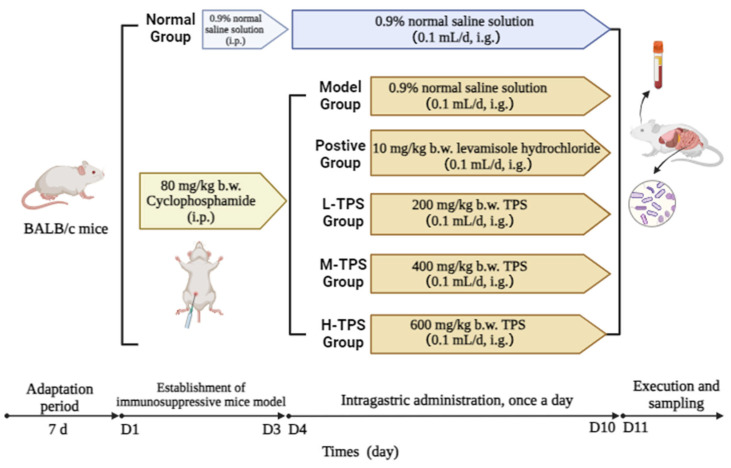
The experimental protocol and drug administration procedure.

In accordance with the method of Hong et al. [25], with minor modifications, forty-eight BALB/c mice were fed and provided water ad libitum for 7 days at room temperature (22 °C ± 2 °C) and 60% ± 5% humidity under a 12 h day–night cycle for acclimation. Next, they were randomly divided into six groups (eight replicates in each group; Figure 1): normal group (NG), model group (MG), positive group [PG; 10 mg/kg body weight (b.w.) LH], low-dose TPS group (L-TPS; 200 mg/kg b.w. TPSs), medium-dose TPS group (M-TPS; 400 mg/kg b.w. TPSs), and high-dose TPS group (H-TPS; 600 mg/kg b.w. TPSs).

Mice in all groups except NG were intraperitoneally injected with CTX at 80 mg/kg b.w. for 3 consecutive days to establish immunosuppression. In contrast, NG mice were intraperitoneally injected with normal saline. After the successful establishment of the mouse model, all animals except those in the NG and MG were administered 0.1 mL of the appropriate test solution via gavage at 9 a.m. daily for 7 consecutive days. In contrast, the NG and MG mice were administered 0.1 mL of normal saline via gavage at 9 a.m. daily for 7 consecutive days.

### 2.4. Weight Change Recording

During the experiments, the body weight of each group of mice was measured daily before gavage; mouse growth and states were also observed.

### 2.5. Sample Collection and Organ Index Determination

After the final gavage, all mice underwent a fasting period of 24 h. Next, they were weighed. We also collected their blood from the posterior orbital venous plexus into an anticoagulant-coated vacuum collection tube. The blood was centrifugated at 4000 rpm and 4 °C for 10 min to obtain plasma, which was stored at −80 °C until use for immune index evaluation.

At the end of the treatment, all mice were euthanized via cervical dislocation. Their thymuses were weighed, and their thymus indexes were calculated as thymus weight (mg)/body weight (mg) × 100%. Thymus tissue was also fixed in 4% paraformaldehyde for histopathological analysis. Spleen tissue was also snap-frozen in liquid nitrogen and stored at −80 °C until use for spleen tissue immune index evaluation. Cecal contents from the mice were obtained under sterile conditions and subsequently transferred into sterile cryogenic tubes. These samples were then snap-frozen in liquid nitrogen and stored at −80 °C until use in gut microbiota sequencing. Finally, the colon contents of the mice were also collected, placed into cryopreservation tubes, and subsequently stored at −80 °C for future application in metabolomic sequencing.

### 2.6. Histopathological Observation

The fixed tissues were dehydrated with a gradient ethanol solution, embedded in paraffin, sectioned, and stained with H&E staining. After staining, the slices were sealed with neutral gum and the pathological changes in the tissues were observed under a microscope.

### 2.7. IgA, IgM, TNF-α, IL-6, IL-1β, and iNOS Quantification in Mouse Plasma and Spleen

To 20 mg blocks of frozen spleen tissue, we added precooled phosphate-buffered saline at a ratio of 1:9 (*w*/*v*). After homogenization, the homogenate was centrifuged at 15,000 rpm at 4 °C for 10 min and the supernatant was collected and centrifuged again. The contents of TNF-α, IL-6, IL-1β, and iNOS in the homogenate supernatant, as well as in the plasma, were determined according to the manufacturer’s instructions for the ELISA kits.

### 2.8. DNA Extraction and 16S rDNA Gene Sequencing

Mouse cecal contents were subjected to 16S rDNA high-throughput sequencing. First, HiPure Stool DNA Kits (model D3141; Guangzhou Megi Biotechnology, Guangzhou, China) were used to extract total bacterial DNA according to the manufacturer’s instructions. Qualified DNA samples were then used to amplify the V3-V4 region of 16S rDNA through the polymerase chain reaction; the following primer sequences were used: 341F (CCTACGGGNGGCWGCAG) and 806 R (GGACTACHVGGGTATCTAAT) [26]. Next, the purified amplicons were connected to a sequencing adapter to construct a sequencing library, which was then sequenced on an Illumina PE250 by Guangzhou Gideo Biotechnology (Guangzhou, China). The analysis results were based on sequencing reads and operational taxonomic units.

### 2.9. Metabolite Extraction for Nontargeted Metabolomics

The metabolomic analysis process has been reported [27]. Mouse colonic contents were analyzed through the UPLC-Q-TOF/MS/MS method for untargeted metabolomics—including metabolite extraction, liquid chromatography–tandem mass spectrometry (LC-MS/MS) analysis, metabolite identification, differential metabolite screening, and bioinformatic analysis.

#### 2.9.1. Metabolite Extraction

The frozen sample was thawed gradually at 4 °C and ground in a grinder (30 Hz, 1.5 min). Next, 100 mg of the ground sample was added to 1 mL methanol/acetonitrile/water solution (2:2:1, *v*/*v*/*v*), followed by vortex mixing and sonication at a low temperature for 30 min. The mixture was allowed to stand at −20 °C for 10 min, followed by centrifugation at 14,000 rpm at 4 °C for 20 min. Subsequently, the supernatant was subjected to vacuum drying. For mass spectrometry (MS) analysis, the dried powder was redissolved in 100 μL of aqueous acetonitrile solution (acetonitrile: water = 1:1, *v*/*v*) with vortexing, followed by centrifugation at 14,000 rpm at 4 °C for 15 min. Finally, the supernatant was filtered through a 0.22-μm filter membrane and subjected to LC-MS/MS.

#### 2.9.2. LC-MS/MS Conditions

The samples were measured using an Agilent 1290 Infinity LC system, followed by MS on an AB Triple TOF 6600 mass spectrometer. The chromatography conditions were as follows: chromatographic column, ACQUITY UPLC BEH Amide column (1.7 μm, 2.1 mm × 100 mm; Waters, Ireland); mobile phase A, water + 25 mmol/L ammonium acetate + 25 mmol/L ammonia; mobile phase B, acetonitrile; flow rate, 0.5 mL/min; injection volume, 2 μL; gradient elution. Moreover, the MS conditions were as follows: ion source gas1 (Gas1), 60 psi; Gas2, 60 psi; curtain gas, 30 psi; source temperature, 600 °C; ionspray voltage floating, ±5500 V; primary mass-to-charge ratio detection range, 60–1000 Da; mass-to-charge ratio of second-order daughter ions, 25–1000 Da; cumulative scanning time of first-order MS, 0.20 s/spectra; cumulative scanning time of secondary MS, 0.05 s/spectra; secondary MS modes, data-dependent acquisition and peak intensity value screening mode; cluster voltage, ±60 V; collision energy, 35 ± 15 eV; dynamic isotope ion exclusion range, 4 Da; number of fragment patterns collected per scan, 10. 

#### 2.9.3. Data Analysis

The differential metabolites were screened and identified using multivariate statistical analysis. The multivariate statistical analysis methods included partial least squares discrimination analysis (PLS-DA) and orthogonal partial least squares discrimination analysis (OPLS-DA). We used MBRole (versions 2.0) and MetaboAnalyst (versions 5.0) for the Kyoto Encyclopedia of Genes and Genomes (KEGG) pathway enrichment analysis (https://www.genome.jp/kegg/, (accessed on 18 August 2024)), with *p* < 0.05 set as a standard screening for affected major metabolic pathways.

### 2.10. Statistical Analysis

All data, expressed as mean ± standard deviation, were processed and analyzed in MS Excel (version 2022). SPSS (version 21; IBM) was used for one-way analysis of variance, and the least significant difference method was used for multiple comparisons for evaluating the significance level (*p* < 0.05). Finally, Origin (version 2022) was used for mapping.

## 3. Results and Discussion

### 3.1. Effects of TPSs on Body Weight in Mice with CTX-Induced Immunosuppression

**Figure 2:**
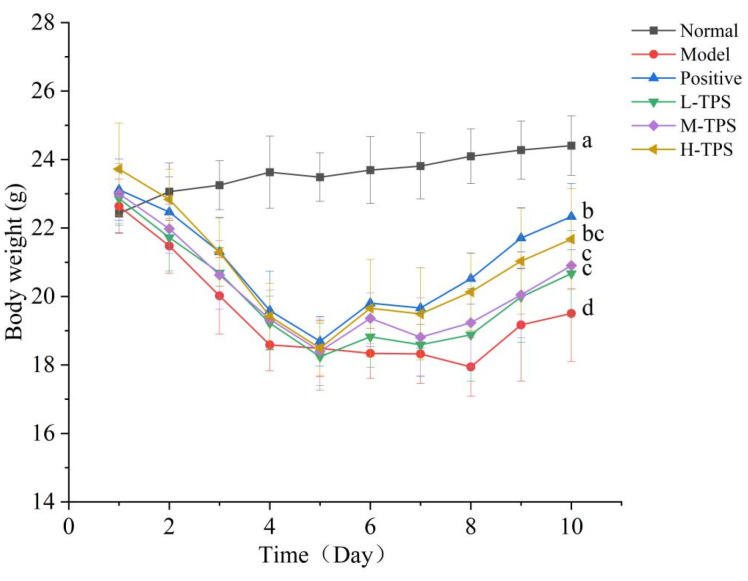
Effects of TPSs on body weights in immunosuppressed mice. A one-way ANOVA test was used for significance analysis, with statistically significant differences among the groups indicated by distinct letters (*p* < 0.05).

Weight is the most intuitive indicator of health. Weight loss can inhibit the development, maturation, and metabolism of immune organs, resulting in a decrease in the immune response. As shown in Figure 2, intraperitoneal CTX injection over 3 consecutive days led to a significant decrease in mouse body weight (*p* < 0.05). Next, gavage feeding of TPSs over 7 consecutive days led to an increase in mouse body weight at varying degrees; the degree of body weight recovery was positively correlated with the TPS dose. Compared with that of MG mice, the body weight of the L-TPS, M-TPS, and H-TPS increased significantly (*p* < 0.05); in these groups, we noted a 5.99%, 7.18%, and 11.11% increase in body weight, respectively. Thus, TPSs facilitated effective body weight recovery in mice with CTX-induced immunosuppression.

### 3.2. Effects of TPSs on Thymus Tissue in Mice with CTX-Induced Immunosuppression

**Figure 3:**
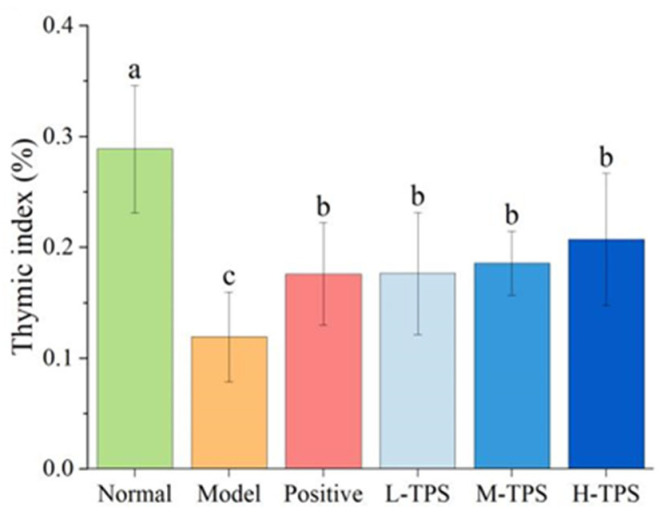
Effect of TPSs on the organ indexes of thymuses in immunosuppressed mice. One-way ANOVA test was used for significance analysis, with statistically significant differences among the groups indicated by distinct letters (*p* < 0.05).

**Figure 4:**
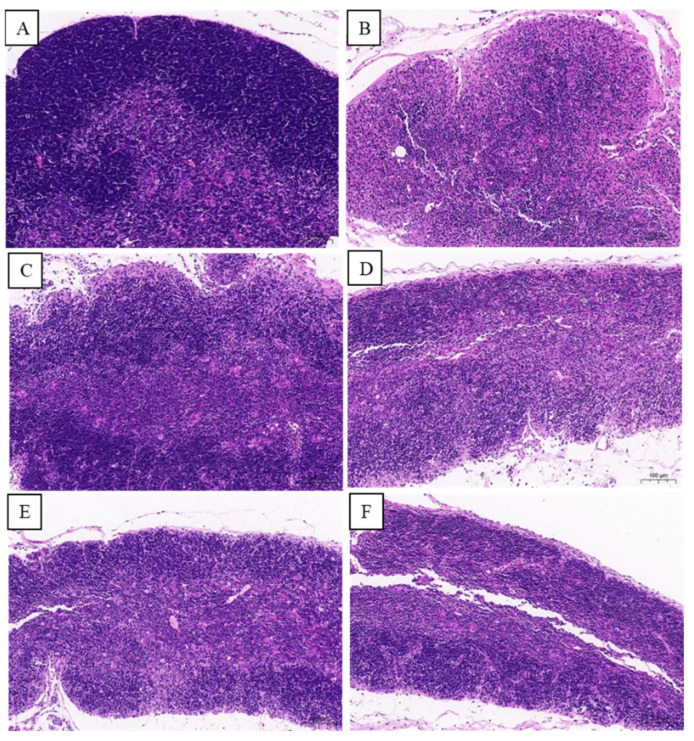
Sectional images of mice thymus (magnification, 200×). (**A**) NG; (**B**) MG; (**C**) PG; (**D**) L-TPS; (**E**) M-TPS; (**F**) H-TPS.

Immune organs play a role in protecting the body from infection, and their immunomodulatory activity is closely associated with changes in the immune organ index. The thymus, a central immune organ, plays a crucial regulatory role in peripheral immune organs and immune cells [28]. Immune organ indicators can somewhat reflect immune function. In this study, the thymus index of MG was significantly lower than that of NG (*p* < 0.05; Figure 3). The results of H&E staining (Figure 4) demonstrated that, in the MG, the thymus was atrophic, with an obscure boundary between the cortex and medulla and a significant reduction in small lymphocyte counts; therefore, CTX can cause thymus atrophy and reduces the numbers of lymphocytes in the tissues. This result is consistent with that of Zhao et al. [29].

Compared with MG mice, PG, L-TPS, M-TPS, and H-TPS mice had a significantly higher thymus index (*p* < 0.05); moreover, PG and H-TPS mice had slightly fewer endolymphocytes, with a more obvious boundary between the cortex and medulla. These results demonstrated that, in mice, CTX can induce thymus atrophy, which can be alleviated by LH and TPSs. These findings, consistent with those noted for *Pleurotus eryngii* polysaccharides [30], suggest that TPSs can improve immune function by reversing CTX-induced immune organ and tissue damage.

### 3.3. Effects of TPSs on Plasma IgA, IgM, TNF-α, IL-6, IL-1β, and iNOS in Mice with CTX-Induced Immunosuppression

**Figure 5:**
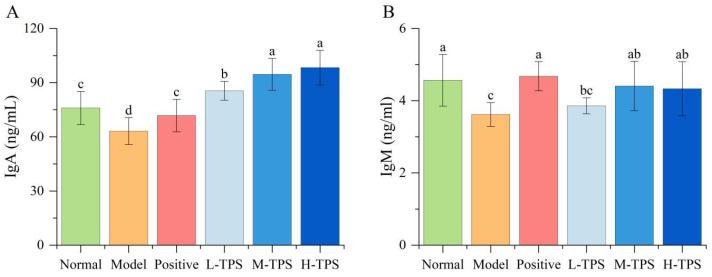
Effects of TPSs on plasma (**A**) IgA and (**B**) IgB levels in immunosuppressed mice. A one-way ANOVA test was used for significance analysis, with statistically significant differences among the groups being indicated by distinct letters (*p* < 0.05).

**Figure 6:**
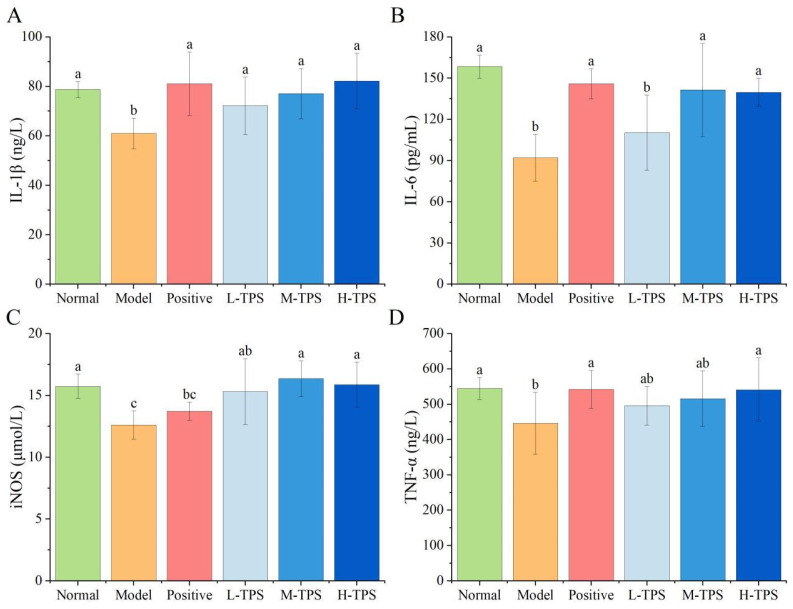
Effects of TPSs on plasma (**A**) IL-1β, (**B**) IL-6, (**C**) iNOS, and (**D**) TNF-α levels in immunosuppressed mice. A one-way ANOVA test was used for significance analysis, with statistically significant differences among the groups being indicated by distinct letters (*p* < 0.05).

In the current study, we measured the plasma levels of immune factors in immunosuppressed mice using ELISA. As shown in Figure 5 and Figure 6, compared with NG mice, MG mice demonstrated significantly lower plasma IgA, IgM, IL-1β, IL-6, iNOS, and TNF-α levels (*p* < 0.05), indicating that CTX can significantly inhibit immune factor secretion in mice.

CTX can reduce the plasma cytokine or Ig levels, lowering immune function [31,32]. In this study, plasma IL-6, IL-1β, TNF-α, IgA, and IgM levels were relatively low in MG mice, indicating the effectiveness of CTX as an immunosuppressant [33]. Compared with MG mice, PG, L-TPS, M-TPS, and H-TPS mice had significantly higher plasma IgA levels (*p* < 0.05); moreover, the plasma IgA levels were significantly higher in L-TPS, M-TPS, and H-TPS mice than in PG mice. Moreover, plasma IL-1β levels significantly increased in PG, L-TPS, M-TPS, and H-TPS mice (*p* < 0.05), and plasma TNF-α levels significantly increased in PG and H-TPS mice (*p* < 0.05). These results suggested that both LH and TPSs significantly enhance cytokine and iNOS secretion in immunosuppressed mice; at moderate and high doses, TPSs had superior effects.

### 3.4. Effects of TPSs on Spleen IgA, IgM, TNF-α, IL-6, IL-1β, and iNOS in Mice with CTX-Induced Immunosuppression

**Figure 7:**
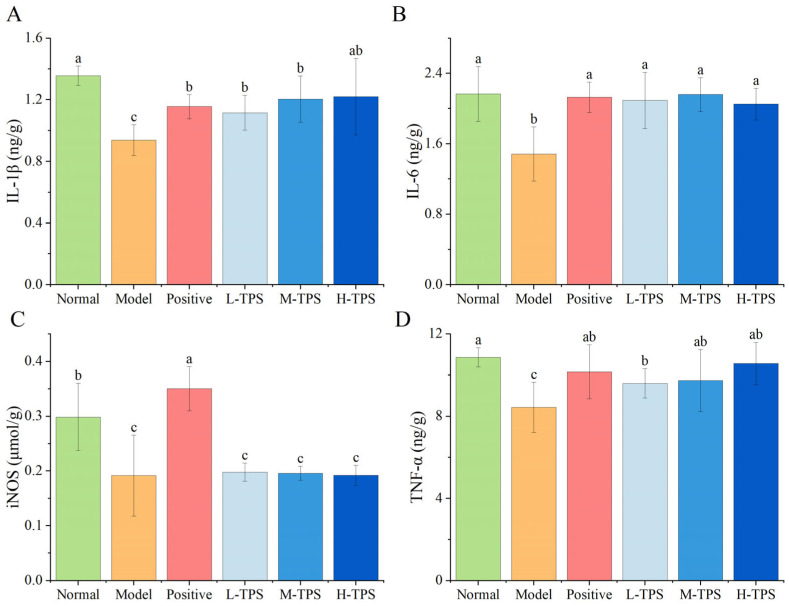
Effects of TPSs on spleen (**A**) IL-1β, (**B**) IL-6, (**C**) iNOS, and (**D**) TNF-α levels in immunosuppressed mice. A one-way ANOVA test was used for significance analysis, with statistically significant differences among the groups being indicated by distinct letters (*p* < 0.05).

Next, we assessed the effects of TPSs on cytokines and iNOS secretion in the spleen of immunosuppressed mice. As shown in Figure 7, compared with NG mice, MG mice demonstrated significantly lower spleen IL-1β, IL-6, iNOS, and TNF-α levels (*p* < 0.05), indicating that CTX can significantly inhibit the cellular and humoral immunity levels of the mice—consistent with the results noted for plasma cytokine levels. Compared with MG mice, PG, L-TPS, M-TPS, and H-TPS mice demonstrated significantly higher spleen IL-1β, IL-6, and TNF-α levels (*p* < 0.05); however, no significant differences were noted in the spleen iNOS levels (*p* > 0.05). These results indicated that TPSs significantly increased IL-1β, IL-6, and TNF-α levels in the spleens of immunosuppressed mice, further demonstrating that TPSs can enhance cellular and humoral immunity in mice and that TPSs may improve systemic immune function by increasing the secretion level of cytokines and immunoglobulins—consistent with the results of Hwang et al. [34].

Studies have reported that many plant polysaccharides can alleviate CTX-induced immune deficiency by increasing cytokine production. However, the mechanisms underlying these effects may be complex. In vivo, polysaccharides induce signal transduction in immune cells through Toll-like receptors 2 and 4; then, they induce cytokine secretion and enhance immune response [35]. Moreover, polysaccharides regulate host immunity by regulating the gut microbiota and promoting intestinal immune function [22]. However, the specific mechanisms underlying the effects of polysaccharides on cytokine secretion warrant further elucidation. Furthermore, certain researchers have found that polysaccharides exhibiting elevated levels of Ara and Gal demonstrate enhanced immunomodulatory properties [36,37]. In the present study, the molar percentages of Gal and Ara in the total polysaccharide content of TPSs constituted 70.25%, a notably high proportion. This finding may be associated with the pronounced immunoenhancing effects attributed to TPSs.

### 3.5. Effects of TPSs on Gut Microbiota in Mice with CTX-Induced Immunosuppression

The gut, rich in microorganisms, is the largest digestive and immune organ. Through long-term interaction with the host, the gut forms a microbiota–host symbiotic relationship. The ability of gut microbiota to adapt to the host’s needs through various immune and metabolic pathways may largely determine health status and response to diseases in the host, and regulation of the gut microbiome composition may effectively inhibit the occurrence and development of various diseases. Accumulating evidence has confirmed the presence of a strong interaction between the complex intestinal ecosystem and the host immune system [38,39]. Therefore, in many studies on the health effects of PPSs, the gut microbiota has been considered a vital target. The plant polysaccharide–gut microbiota interaction and its effect on host immune homeostasis may explain the mechanism underlying various effects of PPSs.

**Figure 8:**
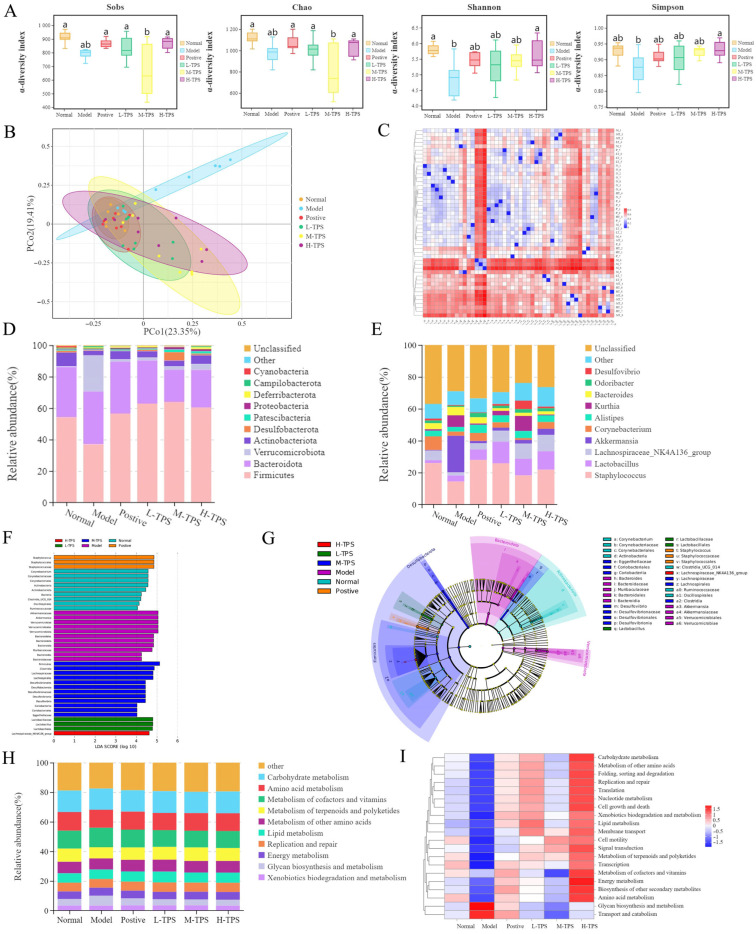
Effects of TPSs on gut microbiota in immunosuppressed mice. (**A**) Alpha diversity indexes. Tukey’s HSD test was used for significance analysis, with statistically significant differences among the groups being indicated by distinct letters (*p* < 0.05). (**B**) PCoA plot. (**C**) Heatmap based on the Bray–Curtis distance. (**D**) Stacked map of species distribution at the phylum level. (**E**) Stacked map of species distribution at the genus level. (**F**) Indicator analysis results. (**G**) Linear discriminant analysis effect size analysis results (LAD > 4.0). (**H**) Stacked plot of functional analysis results. (**I**) Heatmap of functional distribution.

Here, we obtained 5008 high-quality bacterial 16S rDNA gene reads through sequencing of six sets of mouse stool samples. As shown in Figure 8A, compared with NG mice, MG mice demonstrated significantly lower values of the alpha diversity indexes (Sobs, Chao, Shannon, and Simpson indexes) for the gut microbiota (*p* < 0.05), indicating that CTX reduced gut microbiota diversity, richness, and evenness. Compared with MG mice, L-TPS, M-TPS, and H-TPS demonstrated no significant differences in the Sobs and Chao indexes (*p* > 0.05); nevertheless, H-TPS mice demonstrated significantly higher Shannon and Simpson indexes than MG mice (*p* < 0.05), reaching levels similar to those in NG mice. This result indicated that TPSs enhanced gut microbiota diversity in immunosuppressed mice.

In the principal coordinate analysis (PCoA; Figure 8B), PCoA1 and PCoA2 contributed 23.35% and 19.41% to the sample, respectively. In the PCoA plot, the distance between the MG and other (NG, PG, and TPS) groups differed significantly, indicating that gut microbiota composition and structure differed between the MG mice and those from the other groups. In Figure 8C, the color of the cross points between the samples of TPS-treated mice (L-TPS, M-TPS, and H-TPS mice), PG, and NG is generally light, with the difference distances being similar. Therefore, the gut microbiota in TPS- or LH-treated mice were similar to that in NG mice. This result also indicated that TPSs effectively reversed CTX-induced disorder in the gut microbiota in the mice. Moreover, the bacterial flora structure was restored to its normal level. These results are consistent with those noted for Fu brick TPSs [40] and *Camellia* polysaccharides [41].

As shown in Figure 8D, Firmicutes and Bacteroidetes were the dominant bacterial phyla in all groups. Compared with NG mice, MG mice demonstrated a significantly lower relative abundance of Firmicutes and Actinobacteria (*p* < 0.05) but a significantly higher relative abundance of Verrucomicrobiota (*p* < 0.05). Therefore, CTX may have led to an imbalance in Verrucomicrobiota abundance, which then resulted in immune response initiation. In contrast, TPSs significantly lowered the relative abundance of Verrucomicrobiota (*p* < 0.05) to levels similar to those in NG mice, suggesting that TPSs regulated the gut microbiota structure and composition in mice and facilitated the restoration of gut microbiota balance.

As shown in Figure 8E, at the genus level, compared with NG mice, MG mice demonstrated a significantly lower relative abundance of *Staphylococcus* and *Corynebacterium* (*p* < 0.05) but a significantly higher relative abundance of *Akkermansia* (*p* < 0.05). *Akkermansia*, a potential probiotic belonging to the phylum Verrucomicrobiota, mainly uses intestinal mucin as the sole carbon and nitrogen source for growth. *Akkermansia* can regulate the body’s metabolic balance and immune tolerance but also improve intestinal metabolite production and increase the thickness of the intestinal mucosal layer, which has a protective effect on the intestinal mucosa [42,43]. However, the nutritional environment of the host can affect *Akkermansia* growth in the gut, and the mucin degradation property of *Akkermansia* can be a competitive advantage in an undernourished host [42]. Thus, after CTX-induced malnutrition, the immunosuppressed mice demonstrated a significant increase in *Akkermansia* abundance.

Compared with MG mice, PG, L-TPS, M-TPS, and H-TPS mice demonstrated a significant reduction in the relative abundance of *Akkermansia* (*p* < 0.05); however, the relative abundance of *Akkermansia* did not differ significantly between PG and NG mice. Notably, compared with NG mice, L-TPS, M-TPS, and H-TPS mice demonstrated an increase in the relative abundance of the *Lactobacillus* and *Lachnospiraceae* bacterium NK4A136 group (hereafter, L. group) to varying degrees. In particular, L-TPS mice demonstrated a significant increase in the relative abundance of *Lactobacillus* (*p* < 0.05), and M-TPS and H-TPS groups demonstrated a significant increase in the relative abundance of the L. group (*p* < 0.05).

To determine the key genera and potential mechanisms underlying the immunomodulatory effects of TPSs, we performed a linear discriminant analysis (Figure 8F,G) and identified specific bacteria associated with the effects of TPSs. *Lactobacillus*, Firmicutes, and the L. group were noted to be the significantly different species in L-TPS, M-TPS, and H-TPS mice, respectively. The L. group, one of the main genera present in the intestinal tract of mice, might be a beneficial bacterium belonging to the anaerobic, sporogenic bacteria group that can produce SCFAs through fermentation of dietary polysaccharides [44]. Firmicutes possess numerous genes that facilitate the fermentation of dietary fiber and may also engage with the intestinal mucosa to support homeostasis [45]. *Lactobacillus*, a crucial probiotic with adjuvant activity, might be a mucosal vaccine carrier that can reduce the risk of digestive diseases, alleviate lactose intolerance, reduce allergic reactions, increase anticancer activity, and regulate the immune system [46]. Many of the specific biological effects of *Lactobacillus* are mediated through immune regulation, particularly through helper T 1/2-cell and regulatory T-cell balance [47]. Taken together, these results suggest that TPSs regulate the immune status of the body by regulating the gut microbiota structure.

We used PICRUSt2 to predict the functional composition of the gut microbial community by using the 16S rDNA gene sequencing results (Figure 8H,I). The results demonstrated that, compared with MG mice, H-TPS mice demonstrated enhancements in 14 functional pathways. The five most significantly enhanced elements were carbohydrate metabolism, amino acid metabolism, terpenoid and polyketide metabolism, other amino acid metabolism, and lipid metabolism—all are closely related to the repair of CTX-induced tissue damage, promotion of cytokine production and secretion, and activation of signal pathways. These results indicated the mechanisms underlying TPS-induced immune regulation of gut microbiota in immunosuppressed mice.

In summary, CTX can lead to disorder in the composition of mouse gut microbiota, but interventions based on LH or TPSs can regulate this composition, facilitating its transformation to a composition similar to that noted in NG mice. Thus, TPSs play a vital role in gut microbiota regulation.

### 3.6. Metabolomic Analysis of TPS-Treated Mice with CTX-Induced Immunosuppression

**Figure 9:**
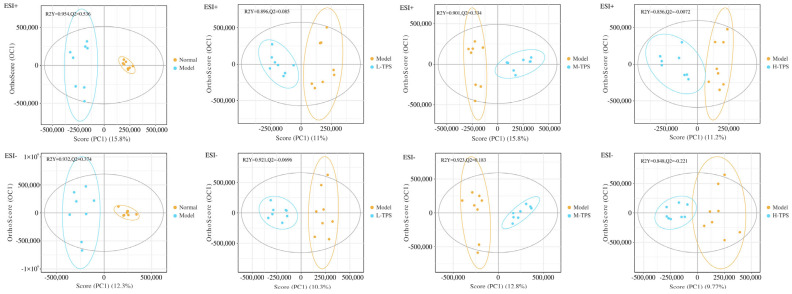
OPLS-DA scores under positive and negative modes.

In our metabolite identification analysis, we detected 2685 and 1655 metabolites in positive and negative ion modes, respectively. To investigate changes in metabolites induced by TPS interventions, relevant data were subjected to a multivariate analysis. Supervised OPLS-DA was used to further distinguish the between-group differences in metabolic profiles in both the positive and negative ion modes. The results demonstrated that the profiles for NG mice were significantly separated from those for MG mice, indicating that CTX induced significant changes in endogenous metabolites in mice. Our PCoA1 plot (Figure 9) demonstrated a clear separation between the MG and experimental (PG and TPS) groups, indicating that the LH and TPS interventions significantly altered endogenous metabolites in the immunosuppressed mice.

**Figure 10:**
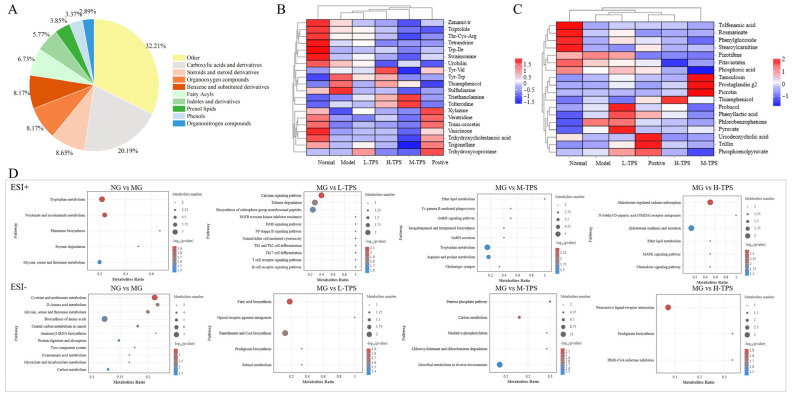
Effect of TPSs on gut metabolites in immunosuppressed mice. (**A**) Pie chart of differential metabolite classification (n = 208). (**B**,**C**) Cluster analysis of differential metabolites in positive (**B**) and negative (**C**) modes. (**D**) KEGG pathway enrichment analysis of differential metabolic pathways in positive and negative ion modes (*p* < 0.05).

Next, we assessed the effects of a TPS intervention on the endogenous substance metabolism in immunosuppressed mice and considered metabolites with variable importance in projection > 1.0, *p* < 0.05 and fold change > 3 to be differential. As shown in Figure 10A, MG mice demonstrated 341 differential metabolites compared with mice in other groups. In total, 208 differential metabolites were classified into 56 categories using MetaboAnalyst, which mainly included carboxylic acids and their derivatives, steroids and their derivatives, organic oxygen compounds, benzene and substituted derivatives, and fatty acyl and indole and their derivatives. CTX treatment led to significant changes in endogenous metabolites compared with NG. The results of the differential metabolite cluster analysis (Figure 10B,C) demonstrated that, compared with MG mice, L-TPS, M-TPS, and H-TPS mice demonstrated significant changes in endogenous metabolites, and they had differential metabolites at levels closer to those in NG mice. These results suggested that TPSs alleviated CTX-induced disorder in metabolisms by modifying intestinal metabolites, with a medium dose of TPSs (400 mg/kg b.w.) demonstrating the highest effectiveness.

To further explore the metabolic pathways of potential biomarkers related to TPS treatment in immunosuppressed mice, we used the R package ClusterProfiler and Enrichplot for performing pathway enrichment analysis of differential metabolites. As shown in Figure 10D, compared with no treatment, CTX treatment led to a significant increase in levels of metabolites related to tryptophan metabolism (2-aminophenol, indole-3-acetamide, and quinolinic acid); niacin and nicotinamide metabolism (L-aspartate and propionic acid); glycine, serine and threonine metabolism (L-aspartate, DL-threonine, and glycine); and other pathways. In contrast, cysteine and methionine (L-glutathione and L-cysteine) metabolism was significantly downregulated. This result suggested that CTX treatment led to significant changes in amino acid metabolism pathways, as well as the abnormal enrichment of some amino acid metabolites. This may have occurred because of CTX-induced low immune statuses, food intake reduction, and insufficient nutrient intakes in mice [48].

In contrast, TPSs were noted to reduce the levels of amino acid metabolites by regulating tryptophan, arginine, and proline metabolism, thereby reversing the abnormal accumulation of amino acid metabolites in the intestines. Tryptophan, an essential aromatic amino acid, can be utilized as a substrate by gut microbiota, immune cells, and the intestinal lining [49]. Under the action of gut microbiota, three main tryptophan metabolic pathways can occur; they involve serotonin (5-hydroxytryptamine), kynurenine, or indole derivatives [50]. In our immunosuppressed mice, CTX induced an increase in indole-3-pyruvate and indole-3-acetamide. After treatment with TPSs, indole-3-pyruvate and indole-3-acetamide levels decreased, indicating that TPSs could regulate the metabolic pathways involving indole-3 derivatives. The findings suggest that TPSs can downregulate the production of indole derivatives in the tryptophan metabolic pathway by regulating the gut microbiota structure.

### 3.7. Analysis of Correlation between Gut Microbiota and Amino Acid Pathway Metabolites

**Figure 11:**
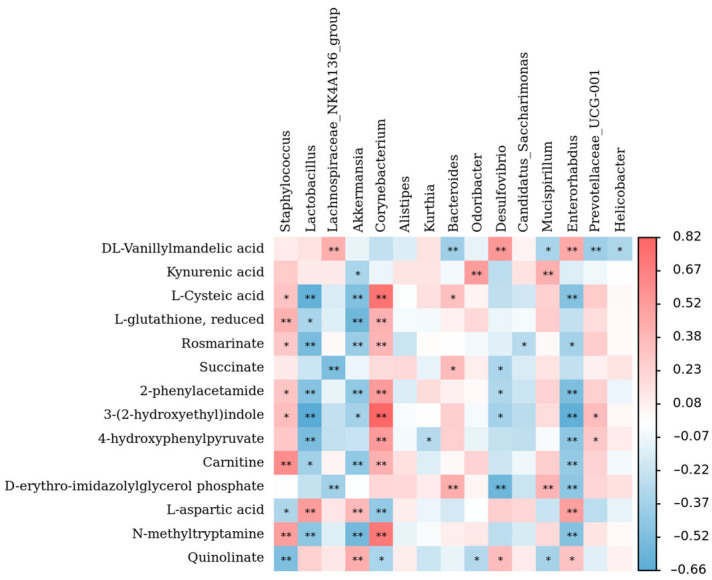
Correlation analysis between gut microbiota and their metabolites at the genus level. * *p* < 0.05, ** *p* < 0.01.

The KEGG pathway enrichment analysis indicated that the immunomodulatory effects of TPSs in immunosuppressed mice were primarily associated with the regulation of amino acid metabolism. As a result, we employed a Spearman correlation analysis to investigate the association between gut microbiota and metabolites associated with the amino acid metabolic pathway. As shown in Figure 11, *Lactobacillus*, *Akkermansia*, and *Enterobacter* were significantly negatively correlated with amino acid metabolism [e.g., metabolism of L-cysteine acid, 2-phenylacetamide, 3-(2-hydroxyethyl) indole, carnitine, N-methyltryptamine; *p* < 0.05]. In contrast, *Corynebacterium* was significantly positively correlated with amino acid metabolism (*p* < 0.05). Thus, the gut microbiota may be closely associated with amino acid pathway-related metabolites. Based on this premise, we propose that TPSs may modulate intestinal mucosal immunity through the microbial–metabolic axis. It seems to exert regulatory effects on the characteristic intestinal microbiota, which may subsequently improve intestinal metabolic disorders and reduce the abnormal accumulation of metabolites. Nonetheless, there remains a deficiency in comprehensive research and analysis regarding the direct interactions between intestinal microbiota and amino acid metabolites, as well as their relationship with the onset and management of diseases associated with immunosuppression. Future investigations are warranted to further explore this area of study.

## 4. Conclusions

In summary, TPSs exhibit immunomodulatory activities by promoting an increase in body weight, stimulating the secretion of immunoglobulins and immune factors, and mitigating thymic damage in mice treated with CTX. Furthermore, TPSs exert a regulatory influence on the dysbiosis of intestinal microbiota caused by CTX and alleviate metabolic disturbances in mice by modulating amino acid metabolism pathways. In the current study, we explored host–gut microbiota interactions and confirmed that TPSs from fresh tea leaves improve gut health and enhance immune function through gut microbiota regulation. These findings provide novel insights into the immunosuppression-alleviating effects of PPSs, which may contribute to the development of newer immunomodulatory strategies and provide a theoretical basis for developing polysaccharide-based functional foods and drugs. 

## Data Availability

The original contributions presented in the study are included in the article, further inquiries can be directed to the corresponding authors.
